# Altered long-range visual hierarchical connectivity in internet gaming disorder: reduced V1–LOC connectivity and its association with addiction severity

**DOI:** 10.3389/fpsyt.2026.1883842

**Published:** 2026-09-11

**Authors:** Jiawen Tian, Hui Zhang, Xinyu Wang, Hongyu Zhang, Longyao Ma, Bohui Mei, Mengzhe Zhang, Yan Lang, Yarui Wei, Shaoqiang Han, Qingqing Lv, Yong Zhang

**Affiliations:** 1 Department of Magnetic Resonance Imaging, the First Affiliated Hospital of Zhengzhou University, Zhengzhou, China; 2 Zhengzhou Key Laboratory of Brain Function and Cognitive Magnetic Resonance Imaging, Zhengzhou, China; 3 Henan Engineering Technology Research Center for Detection and Application of Brain Function, Zhengzhou, China; 4 Henan Engineering Research Center of Medical Imaging Intelligent Diagnosis and Treatment, Zhengzhou, China; 5 Henan Key Laboratory of Imaging Intelligence Research, Zhengzhou, China; 6 Henan Engineering Research Center of Brain Function Development and Application, Zhengzhou, China; 7 Department of Psychiatry, The First Affiliated Hospital of Zhengzhou University, Zhengzhou, China; 8 Department of radiology, The Third Affiliated Hospital of Zhengzhou University, Zhengzhou, China

**Keywords:** addiction severity, functional connectivity, internet gaming disorder, lateral occipital cortex, lingual gyrus, resting-state fMRI, visual hierarchy

## Abstract

**Background:**

Neuroimaging studies of internet gaming disorder (IGD) have primarily focused on reward-related and executive-control networks. However, sensory processing of addiction-related cues, particularly visual processing, may also contribute to IGD. Whether the intrinsic hierarchical organization of the visual system is altered remains unclear.

**Methods:**

The study included 35 individuals with IGD and 30 healthy controls (HCs), with an overall age range of 9–33 years. The sample was predominantly composed of adolescents. An *a priori* three-level visual hierarchy model comprising the primary visual cortex (V1), lingual gyrus, and lateral occipital cortex (LOC) was constructed. Functional connectivity (FC) was calculated for the V1–Lingual, Lingual–LOC, and V1–LOC pathways. A hierarchical integration index (HII), derived from V1–LOC and V1–Lingual connectivity, was calculated to assess long-range relative to local visual coupling. Group differences were assessed using MANCOVA with follow-up univariate analyses and FDR correction. Within the IGD group, associations between IAT scores and all visual hierarchical measures were examined using Pearson and covariate-adjusted partial correlations with FDR correction.

**Results:**

V1–LOC connectivity was significantly reduced in the IGD group compared with HCs (HC: 0.364 ± 0.132; IGD: 0.130 ± 0.189; *F*(1,59) = 30.809; FDR-corrected *p* < 0.001; partial η^2^ = 0.343). V1–Lingual and Lingual–LOC connectivity did not show significant group differences, although their effect sizes were small-to-moderate (Cohen’s *d* = 0.32 and 0.40, respectively). The HII was numerically lower in IGD but did not reach statistical significance (*t* = 1.90, *p* = 0.062; Cohen’s *d* = 0.46). Within the IGD group, lower V1–LOC connectivity was associated with higher IAT scores (*r* = −0.479, FDR-corrected *p* = 0.016), and this association remained significant after controlling for age, sex, education, and mean FD (partial *r* = −0.447, FDR-corrected *p* = 0.048).

**Conclusions:**

IGD is associated with altered long-range hierarchical connectivity within the visual system, particularly between early visual cortex and higher-order LOC. These findings provide a sensory-level perspective complementing reward- and control-centered accounts of IGD.

## 1 Introduction

Internet gaming disorder (IGD) is characterized by impaired control over gaming, excessive involvement in gaming activities, and continued gaming despite negative consequences. IGD has been included in the DSM-5 as a condition warranting further study and has received increasing clinical and research attention because of its psychological, behavioral, and social burden (1–3). Cognitive-behavioral and neurobiological models of IGD emphasize craving, reward seeking, impaired self-regulation, and reduced control over gaming behavior (4–7). Consistent with these models, neuroimaging studies have reported abnormalities in reward processing, executive control, salience attribution, and fronto-striatal circuits (8).

The Interaction of Person-Affect-Cognition-Execution (I-PACE) model further conceptualizes specific Internet-use disorders as emerging from interactions among individual predispositions, affective and cognitive responses to situational triggers, cue-reactivity, craving, and reduced executive functioning (4, 5). However, the neural mechanisms of IGD are unlikely to be limited to reward valuation and executive control. Disorder-related cues must first be perceived and processed through sensory systems before they can trigger cue-reactivity, attentional bias, or craving. This raises the possibility that sensory-level processing, particularly visual processing, may represent an important but relatively underexplored component of IGD neurobiology.

For IGD, the visual system may be especially relevant because online games provide highly visual, dynamic, and reinforcement-rich environments. Previous studies have shown that gaming-related visual cues can elicit gaming urges and addiction-related neural responses in individuals with online gaming addiction (9). Individuals with Internet game addiction also show cognitive biases toward game-related pictures, suggesting that gaming-related visual information may be processed differently in this population (10). Evidence from broader addiction research further supports the importance of visual cue processing. Visual cortical activation has been identified as a reliable component of cue-reactivity to addiction-related stimuli, and sensory systems may contribute to addiction-related cue processing beyond classical reward and control circuits (11, 12). Thus, the visual system is unlikely to function merely as a passive sensory input channel. Instead, it may participate in the transformation of disorder-relevant visual stimuli into cues with attentional and motivational salience. These findings suggest that visual information processing may represent a sensory-level pathway through which disorder-related cues are processed and may contribute to attentional bias and craving.

Visual processing is not a unitary sensory process but follows a hierarchical organization, in which early visual areas encode basic sensory features and higher-order visual association regions integrate complex object-related and contextual information. This hierarchical architecture may be particularly relevant to IGD because gaming-related stimuli often contain highly salient visual features that require integration across multiple levels of representation. Importantly, alterations in long-range communication between early visual cortex and higher-order visual association cortex may disrupt the transformation of visual inputs into behaviorally meaningful representations. Therefore, examining hierarchical connectivity within the visual system may provide a framework for understanding how gaming-related cues are processed beyond traditional reward and executive-control networks. Because V1–LOC connectivity represents communication between early sensory encoding and higher-order visual association processing, we hypothesized that intrinsic functional coupling within the visual hierarchy would be altered in IGD, with particular interest in cross-level communication between early and higher-order visual regions.

Emerging evidence also points to visual-system abnormalities in IGD. A recent resting-state network study reported altered functional network connectivity between the dorsal attention network and the visual network across different levels of gaming engagement (13). This finding suggests that the interaction between attentional control and visual processing may change as gaming-related problems progress. Multimodal neuroimaging evidence has further identified the lingual gyrus as a relevant visual node in IGD, showing both structural alterations and disrupted effective connectivity with visual attention-related regions (14). These studies suggest that visual-related regions are involved in IGD neurobiology, but important questions remain. Most previous work has examined the visual system either as a single large-scale network or through isolated regional abnormalities. It therefore remains unclear whether IGD is associated with alterations in the internal hierarchical organization of the visual system.

The hierarchical organization of the visual system offers a useful framework for examining this issue. Visual processing involves a progression from early sensory encoding to more complex perceptual representations. However, the ventral visual pathway is not simply a serial feedforward stream. Instead, it has been conceptualized as a recurrent and interactive occipitotemporal network, in which early visual areas communicate with higher-order temporal and occipital regions through feedforward, feedback, and long-range interactions (15–17). From this perspective, efficient visual perception depends not only on the functional integrity of individual visual regions, but also on coordinated communication across hierarchical levels.

Within this hierarchy, the primary visual cortex (V1) supports early sensory encoding of basic visual features. The lingual gyrus is located within medial occipitotemporal visual regions and may represent an intermediate-level visual processing node. The lateral occipital cortex (LOC), particularly the lateral occipital complex, has been consistently associated with object-related visual representation and object recognition (18, 19). Examining intrinsic functional coupling among V1, lingual gyrus, and LOC may therefore provide a focused approach for characterizing visual hierarchical organization from early sensory processing to higher-order visual representation.

Within this framework, IGD-related visual abnormalities may not involve a uniform disruption of the entire visual pathway. Instead, they may be more pronounced in the coordination between early sensory areas and higher-order visual association cortices. Long-term exposure to highly dynamic and reinforcement-rich gaming environments could bias visual processing toward rapid local sensory encoding, while increasing the need for efficient long-range cross-hierarchical communication. As a result, communication between early visual cortex and higher-order visual regions may be more vulnerable to functional disorganization, whereas adjacent-level coupling may remain relatively preserved. From a broader addiction perspective, disorder-related visual cues can capture attention through stimulus-driven mechanisms and become linked to salience attribution and motivational responses (20, 21). In line with the I-PACE model, altered visual hierarchical integration may therefore represent a sensory-level pathway through which gaming-related cues are more strongly associated with cue-reactivity, attentional capture, and gaming urges. Therefore, alterations in visual hierarchical connectivity may reflect disrupted sensory transformation processes through which gaming-related visual stimuli are converted into higher-order representations relevant to attentional capture and cue-reactivity.

Because gaming-related cues often contain complex object-level information requiring integration from early sensory features to higher-order visual representations, disrupted communication between V1 and LOC may represent a potential sensory-level mechanism linking visual cue perception to maladaptive attentional and motivational responses in IGD. The present study was conducted in a predominantly adolescent sample. Based on this rationale, the present study constructed an *a priori* three-level visual hierarchy model comprising V1, lingual gyrus, and LOC, representing early, intermediate, and higher-order stages of visual processing, respectively. Resting-state functional connectivity was examined for two adjacent-level pathways, V1–Lingual and Lingual–LOC, as well as one long-range cross-hierarchical pathway, V1–LOC. To further characterize the relative balance between long-range and local visual coupling, we calculated a hierarchical integration index (HII), defined as V1–LOC connectivity relative to V1–Lingual connectivity. We hypothesized that individuals with IGD would show reduced long-range V1–LOC connectivity, while adjacent-level connectivity would be relatively preserved. Therefore, we hypothesized that long-range cross-hierarchical communication between early visual encoding and higher-order object representation regions may show greater vulnerability in IGD, whereas adjacent-level visual coupling might remain relatively preserved. We also examined whether the visual hierarchical measures were associated with addiction severity, as measured by Internet Addiction Test scores.

## 2 Methods

### 2.1 Participants

A total of 35 individuals with Internet Gaming Disorder (IGD) and 30 demographically matched healthy controls (HCs) were recruited for the present study. Participants ranged in age from 9 to 33 years, and the sample was predominantly composed of adolescents. The diagnosis of IGD was established by two experienced psychiatrists according to the Diagnostic and Statistical Manual of Mental Disorders, Fifth Edition (DSM-5).The severity of internet gaming disorder was assessed using the Young’s Internet Addiction Test (IAT). All participants were right-handed.

The inclusion criteria for the IGD group were as follows: (1) meeting DSM-5 diagnostic criteria for IGD; (2) IAT score ≥ 50; (3) no history of antipsychotic or neuropathic medication use; (4) no recent use of sedative, hypnotic, analgesic, or narcotic drugs; (5) no history of severe craniocerebral trauma or organic brain disease; and (6) no substance abuse or dependence other than IGD (e.g., nicotine or alcohol). The inclusion criteria for the HC group were as follows: (1) physical and mental health without any neurological or psychiatric disorder; (2) IAT score< 50; and (3) no history of substance abuse or dependence.

The exclusion criteria for all participants were as follows: (1) intellectual disability; (2) contraindications to MRI, such as metal implants, pacemakers, or claustrophobia; (3) pregnancy or lactation; and (4) current or previous psychiatric disorders or hereditary neurological disorders. In the IGD group, symptom severity was further assessed using the Hamilton Anxiety Scale (HAMA) and the Hamilton Depression Scale (HAMD). All participants or their guardians were informed of the study purpose, procedures, contraindications, and possible discomforts before the examination, and written informed consent was obtained. The study was approved by the Ethics Committee of the First Affiliated Hospital of Zhengzhou University and was conducted in accordance with the Declaration of Helsinki. This study used an observational case-control neuroimaging design, and participants were recruited according to predefined inclusion and exclusion criteria. Imaging data were anonymized before preprocessing to minimize potential bias.

### 2.2 MRI data acquisition

MRI data were acquired on a 3.0-T Siemens Prisma scanner (Siemens Healthcare, Erlangen, Germany) equipped with a 64-channel head coil. During scanning, foam pads and earplugs were used to minimize head motion and reduce scanner noise. Participants were instructed to keep their eyes closed, remain awake, stay still, and avoid falling asleep during the scan. After scanning, all participants reported that they had remained awake throughout the acquisition.

High-resolution T1-weighted structural images were acquired using a 3D magnetization-prepared rapid gradient echo sequence with the following parameters: repetition time (TR) = 2300 ms; echo time (TE) = 2.32 ms; flip angle = 9°; matrix size = 256 × 256; slice thickness = 0.9 mm; slice gap = 0 mm; number of slices = 176; voxel size = 0.9 × 0.9 × 0.9 mm^3^; and field of view (FOV) = 240 × 240 mm^2^.

Resting-state functional images were acquired using a gradient-echo echo-planar imaging sequence with the following parameters: TR = 1000 ms; TE = 30 ms; flip angle = 70°; matrix size = 64 × 64; slice thickness = 2.2 mm; slice gap = 0.5 mm; number of slices = 52; voxel size = 2 × 2 × 2.2 mm^3^; FOV = 220 × 220 mm^2^; and 400 whole-brain volumes.

### 2.3 fMRI data preprocessing

Resting-state fMRI data were preprocessed using the advanced edition of the Data Processing Assistant for Resting-State fMRI (DPARSFA, version 5.0; https://rfmri.org/DPABI), based on SPM12 and implemented in MATLAB R2022b (22, 23). The preprocessing steps were as follows: (1) DICOM images were converted to NIfTI format; (2) the first 10 volumes were discarded to allow for signal stabilization; (3) slice timing correction was performed; (4) head motion correction was conducted, and participants with maximum translation > 2 mm or rotation > 2° were excluded; (5) functional images were spatially normalized to Montreal Neurological Institute (MNI) space using the standard SPM12 EPI template and resampled to 3 × 3 × 3 mm^3^ using trilinear interpolation; (6) linear trends were removed; (7) temporal band-pass filtering (0.01–0.08 Hz) was applied; and (8) nuisance covariates, including the Friston 24 head-motion parameters, white matter (WM) signals, and cerebrospinal fluid (CSF) signals, were regressed out (24). WM and CSF signals were included as nuisance regressors to reduce variance associated with non-neuronal physiological fluctuations and other sources of noise. Global signal regression was not performed. To further minimize motion-related effects, volumes with framewise displacement (FD) > 0.2 mm were identified during scrubbing and handled using spline interpolation (22, 25).

### 2.4 Definition of visual hierarchy ROIs

To examine intrinsic hierarchical functional organization within the visual system, we constructed an *a priori* three-level visual hierarchy model comprising the primary visual cortex (V1), lingual gyrus, and lateral occipital cortex (LOC). This framework was motivated by prior work on the ventral visual pathway, which conceptualizes visual processing as involving recurrent interactions among early visual cortex, intermediate occipitotemporal visual regions, and higher-order visual association areas rather than a strictly unidirectional serial processing stream (15–17).

The selection of these three ROIs was based on their representative positions within the visual processing hierarchy. V1 was selected to represent early visual sensory encoding and basic visual feature processing. The lingual gyrus was selected as an intermediate medial occipitotemporal visual region, and recent multimodal neuroimaging evidence has also implicated the lingual gyrus as an important node in IGD-related visual abnormalities (14). LOC was selected to represent higher-order visual processing, given its established role in object-related visual representation and object recognition (18, 19, 26), as well as its retinotopic and object-selective organization (27, 28). Together, V1, lingual gyrus, and LOC formed a three-level model from early visual input to intermediate visual processing and higher-order visual representation, allowing us to examine both adjacent-level connectivity and long-range cross-hierarchical connectivity between early visual cortex and higher-order visual association cortex.

Six spherical regions of interest (ROIs) with a radius of 6 mm were defined in Montreal Neurological Institute (MNI) space. The ROI centers were selected *a priori* to sample representative locations within early, intermediate, and higher-order visual processing regions, rather than being derived from the present data or determined based on current sample-specific results. Anatomical localization of each ROI center was visually inspected on the MNI template and verified with reference to the Automated Anatomical Labeling (AAL) atlas in xjView (29). Atlas information was used for anatomical labeling and verification only, not for deriving ROI centers or for data-driven ROI selection. The anatomical verification and rationale for ROI selection are summarized in **Supplementary Table S1**.

The six spherical ROIs were centered at the following MNI coordinates (x, y, z): V1, left [−10, −80, 8] and right [10, −80, 8]; lingual gyrus, left [−14, −60, −2] and right [14, −60, −2]; and LOC, left [−44, −72, −4] and right [44, −72, −4]. These ROIs were used for subsequent ROI-based functional connectivity analysis.

### 2.5 ROI-based functional connectivity analysis

For each participant, the mean BOLD time series was extracted from each of the six predefined ROIs using the ROI signal extraction module in DPARSFA, a MATLAB-based toolbox for resting-state fMRI data processing and analysis (22, 23). Time series extraction was performed on the unsmoothed preprocessed functional images to minimize spatial blurring and signal mixing between adjacent visual regions. The extracted time series were therefore based on data that had undergone nuisance covariate regression and temporal band-pass filtering, as described above. After removal of the first 10 volumes during preprocessing, each ROI time series contained 390 time points.

Pairwise Pearson correlation coefficients were calculated between ROIs within the same hemisphere to quantify functional connectivity along the predefined visual hierarchy. Specifically, V1–Lingual connectivity was calculated between the left V1 and left lingual gyrus and between the right V1 and right lingual gyrus. Lingual–LOC connectivity was calculated between the left lingual gyrus and left LOC and between the right lingual gyrus and right LOC. V1–LOC connectivity was calculated between the left V1 and left LOC and between the right V1 and right LOC.

For each participant, Pearson correlation coefficients were first transformed into Fisher’s z values to improve normality. Because no lateralized effects were hypothesized *a priori*, left- and right-hemispheric Fisher z values were then averaged to obtain one bilateral pathway-level connectivity measure for each connection type. Thus, three ROI-based functional connectivity measures were obtained for each participant: V1–Lingual, Lingual–LOC, and V1–LOC connectivity.

To further quantify the relative balance between long-range and local visual hierarchical connectivity, a Hierarchical Integration Index (HII) was calculated for each participant as follows:


HII=zFC(V1–LOC)−zFC(V1–Lingual)

where zFC denotes the bilateral Fisher z-transformed connectivity measure. Lower HII values indicate relatively weaker long-range V1–LOC connectivity compared with local V1–Lingual connectivity.

### 2.6 Statistical analysis

Statistical analyses were performed using IBM SPSS Statistics version 26.0 (IBM Corp., Armonk, NY, USA). Continuous demographic and motion-related variables, including age, years of education, and mean framewise displacement (FD), were compared between the IGD and HC groups using independent-samples *t*-tests. For all independent-samples t-tests, homogeneity of variance was assessed using Levene’s test, and Welch’s t-test was applied when the equal-variance assumption was violated.

Group differences in the three ROI-based functional connectivity measures, including V1–Lingual, Lingual–LOC, and V1–LOC connectivity, were assessed using multivariate analysis of covariance (MANCOVA). Group was entered as the fixed factor, while age, sex, years of education, and mean framewise displacement (FD) were included as covariates. Significant multivariate effects were followed by univariate analyses to identify connectivity measures contributing to the overall effect. The Hierarchical Integration Index (HII) was analyzed separately because it was mathematically derived from the connectivity measures and therefore was not an independent variable. For the follow-up univariate analyses, p values were corrected using the Benjamini–Hochberg false discovery rate (FDR) procedure (30). Both uncorrected and FDR-corrected *p* values were reported. Effect sizes for the follow-up univariate analyses were reported as partial *η^2^*, while Cohen’s *d* was additionally calculated from the unadjusted group means using the pooled standard deviation.

To examine whether the primary group difference was robust to potential demographic and motion-related confounds, a sensitivity analysis was conducted using a univariate general linear model (GLM). V1–LOC connectivity was entered as the dependent variable; group and sex were entered as fixed factors; and age, years of education, and mean framewise displacement (FD) were entered as covariates. Only main effects were included in the model.

To further assess the robustness of the ROI-based functional connectivity findings, an additional sensitivity analysis was performed using alternative ROI sizes. The ROI-based functional connectivity analyses were repeated using spherical ROIs with radii of 5 mm and 4 mm, while maintaining the same MNI coordinates, preprocessing pipeline, and statistical procedures as the primary 6-mm ROI analysis. The consistency of group differences across the predefined visual hierarchical connections was evaluated. For clinical associations, the same partial correlation procedure was applied to examine the relationship between V1–LOC connectivity and IAT scores while controlling for age, sex, education, and mean FD.

Within the IGD group, Pearson correlation analyses were performed to examine the associations between IAT scores and all visual hierarchical measures, including V1–Lingual, Lingual–LOC, V1–LOC connectivity, and HII. Partial correlation analyses were subsequently conducted while controlling for age, sex, years of education, and mean FD. To account for multiple testing, Benjamini–Hochberg FDR correction was applied separately across the four visual hierarchical measures for Pearson and partial correlation analyses. Statistical significance was set at two-tailed FDR-corrected *p* < 0.05.

A sensitivity power analysis was performed using G*Power 3.1 for a two-sided independent-samples comparison, based on the current sample sizes (35 individuals with IGD and 30 HCs), an alpha level of 0.05, and 80% statistical power. The analysis indicated a minimum detectable effect size of approximately Cohen’s d = 0.71.

## 3 Results

### 3.1 Demographic and clinical characteristics

Demographic and clinical characteristics of the participants are summarized in **Table 1**. The IGD and HC groups did not differ significantly in age, education, or mean framewise displacement. Specifically, Welch’s *t*-tests showed no significant group differences in age, *t*(33.884) = −1.371, *p* = 0.179, education, *t*(37.825) = −0.732, *p* = 0.469, or mean framewise displacement, *t*(62.324) = 0.660, *p* = 0.511. These results indicate that the two groups were generally comparable in demographic characteristics and head motion. Clinical assessments were available for the IGD group, with mean scores of 63.31 ± 11.54 for IAT, 16.37 ± 10.78 for HAMA, and 23.69 ± 11.36 for HAMD. The IGD group included 26 males and 9 females, whereas the HC group included 23 males and 7 females. There was no significant difference in sex distribution between the two groups (*χ^2^* = 0.049, *p* = 0.824).

**Table 1:** Demographic and clinical characteristics of participants.

Variable	IGD (n = 35)	HC (n = 30)	Statistic	p
Age	14.43 ± 1.93	16.03 ± 6.16	t = −1.370	0.179
Education	8.43 ± 1.69	9.00 ± 3.98	t = −0.730	0.469
Mean FD	0.145 ± 0.049	0.138 ± 0.038	t = 0.660	0.511
IAT	63.31 ± 11.54	—	—	—
HAMA	16.37 ± 10.78	—	—	—
HAMD	23.69 ± 11.36	—	—	—
Sex, male/female	26/9	23/7	χ^2^ = 0.049	0.824

Values are presented as mean ± standard deviation. Independent-samples t-tests were used to compare demographic and head-motion variables between groups. Welch’s t-test results were reported when the homogeneity of variance assumption was violated. IAT, HAMA, and HAMD scores were available only for the IGD group and are therefore reported descriptively. IGD, Internet gaming disorder; HC, healthy controls; IAT, Internet Addiction Test; HAMA, Hamilton Anxiety Rating Scale; HAMD, Hamilton Depression Rating Scale; FD, framewise displacement.

### 3.2 Group differences in visual hierarchical functional connectivity

To examine whether IGD was associated with altered intrinsic visual hierarchical connectivity, a multivariate analysis of covariance (MANCOVA) was performed with V1–Lingual, Lingual–LOC, and V1–LOC connectivity as dependent variables, group as the fixed factor, and age, sex, years of education, and mean framewise displacement (FD) as covariates. The MANCOVA revealed a significant overall effect of group on visual hierarchical connectivity (Wilks’ *λ* = 0.656, *F*(3,57) = 9.950, *p<* 0.001, partial η^2^ = 0.344). Follow-up univariate analyses (**Supplementary Table S2**) indicated that this effect was primarily driven by reduced V1–LOC connectivity in the IGD group (*F*(1,59) = 30.809, *p<* 0.001, partial η^2^ = 0.343), whereas V1–Lingual and Lingual–LOC connectivity did not show significant group differences (*p* > 0.05).

**Figure 1:**
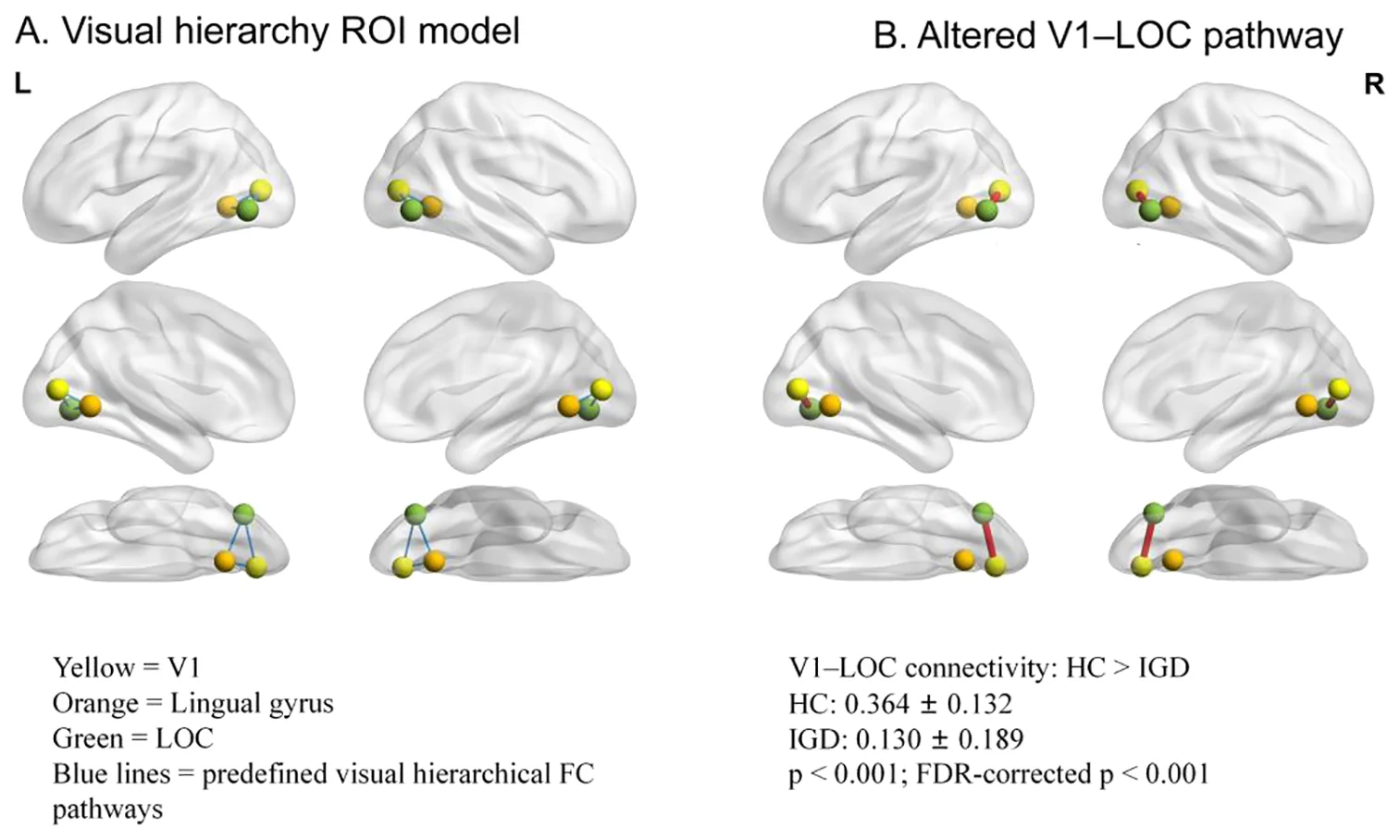
Visual hierarchy ROI model and altered V1–LOC pathway in IGD. **(A)** The predefined visual hierarchy ROI model included bilateral V1, lingual gyrus, and lateral occipital cortex (LOC). Blue lines indicate the examined visual hierarchical functional connectivity pathways, including V1–Lingual, Lingual–LOC, and V1–LOC connectivity. Yellow nodes indicate V1, orange nodes indicate the lingual gyrus, and green nodes indicate LOC. **(B)** The V1–LOC pathway showed significantly reduced functional connectivity in individuals with IGD compared with HCs. Red lines highlight the altered V1–LOC pathway. V1–LOC connectivity was higher in HCs than in individuals with IGD (HC: 0.364 ± 0.132; IGD: 0.130 ± 0.189; *p* < 0.001; FDR-corrected *p* < 0.001). IGD, Internet gaming disorder; HC, healthy controls; LOC, lateral occipital cortex; FC, functional connectivity. Colors are shown for visualization purposes.

The predefined visual hierarchy ROI model and the altered V1–LOC pathway are illustrated in **Figure 1**. Group comparisons revealed that V1–LOC connectivity was significantly lower in individuals with IGD than in HCs, and this difference remained significant after FDR correction (HC: 0.364 ± 0.132; IGD: 0.130 ± 0.189; *t* = 5.69; *p* < 0.001; FDR-corrected *p* < 0.001).

V1–Lingual connectivity showed a nominal uncorrected group effect (F(1,59) = 4.046, p = 0.049), but this effect did not survive FDR correction (FDR-corrected p = 0.073). Lingual–LOC connectivity did not show a significant group difference (F(1,59) = 2.857, p = 0.096, FDR-corrected p = 0.096). In contrast, V1–LOC connectivity remained significantly reduced in the IGD group after FDR correction (F(1,59) = 30.809, FDR-corrected p< 0.001).

**Figure 2:**
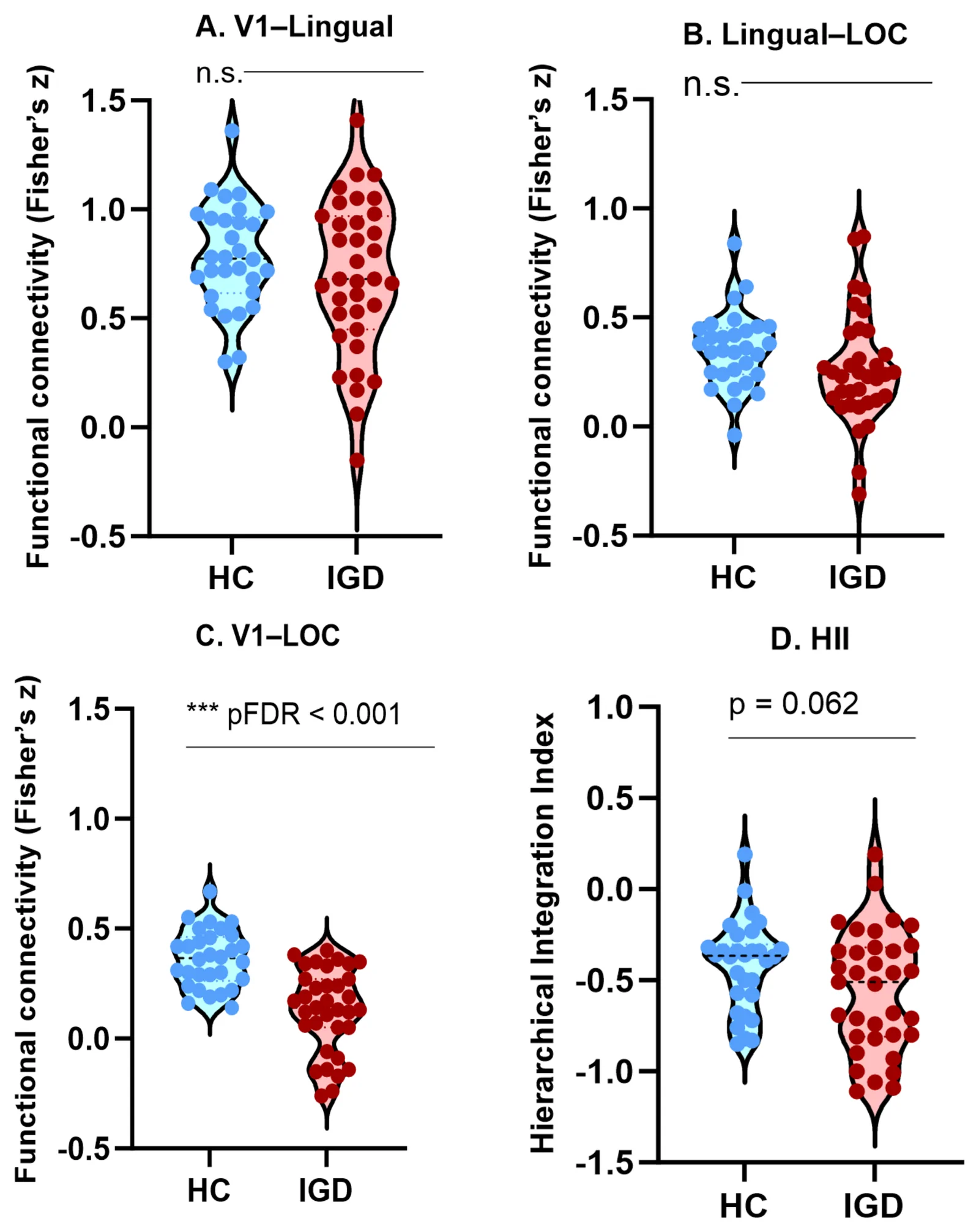
Group differences in visual hierarchical functional connectivity. Group differences are shown for V1–Lingual connectivity, Lingual–LOC connectivity, V1–LOC connectivity, and the Hierarchical Integration Index (HII). Individuals with IGD showed significantly reduced V1–LOC connectivity compared with healthy controls (HCs), whereas V1–Lingual and Lingual–LOC connectivity did not differ significantly between groups. The HII was numerically lower in the IGD group but did not reach statistical significance. Violin plots indicate the distribution of connectivity values, and individual dots represent participant-level data. ***FDR-corrected *p* < 0.001. n.s., not significant; HC, healthy control; IGD, Internet gaming disorder; LOC, lateral occipital cortex; HII, Hierarchical Integration Index.

The HII was numerically lower in the IGD group than in the HC group but did not reach statistical significance (t = 1.90, p = 0.062, Cohen’s d = 0.46).Group differences across the visual hierarchy measures are summarized in **Table 2**; **Supplementary Table S3**, and visualized in **Figure 2**.

**Table 2:** Group differences in visual hierarchical functional connectivity.

Measure	HC mean ± SD	IGD mean ± SD	Statistic	*p*	FDR-corrected *p*	Effect size
V1–Lingual	0.786 ± 0.237	0.689 ± 0.352	*F*(1,59) = 4.046	0.049	0.073	partial η^2^ = 0.064; d = 0.32
Lingual–LOC	0.353 ± 0.172	0.265 ± 0.252	*F*(1,59) = 2.857	0.096	0.096	partial η^2^ = 0.046; d = 0.40
V1–LOC	0.364 ± 0.132	0.130 ± 0.189	*F*(1,59) = 30.809	<0.001	<0.001	partial η^2^ = 0.343; d = 1.42
HII	−0.422 ± 0.248	−0.559 ± 0.332	*t* = 1.90	0.062	—	*d* = 0.46

Values are presented as mean ± standard deviation. Group differences in the three primary ROI-based functional connectivity measures were assessed using MANCOVA, with age, sex, years of education, and mean framewise displacement as covariates. Following the significant multivariate group effect, univariate analyses were performed for V1–Lingual, Lingual–LOC, and V1–LOC connectivity. *P* values for these three follow-up tests were corrected using the Benjamini–Hochberg false discovery rate procedure. Partial η^2^ represents the effect size from the covariate-adjusted univariate analyses, whereas Cohen’s *d* was calculated using the pooled standard deviation. HII was analyzed separately as a secondary derived measure because it was mathematically derived from V1–LOC and V1–Lingual connectivity and was not included in the MANCOVA.

Together, these findings indicate that the most robust group difference was observed in long-range V1–LOC connectivity, rather than reflecting a uniform reduction across all examined visual hierarchical connections.

### 3.3 Robustness analyses and statistical power evaluation

Given the current sample size, the sensitivity power analysis indicated that the study was capable of detecting between-group effects of approximately Cohen’s d ≥ 0.71 with 80% power at a two-tailed alpha level of 0.05. Thus, the study had adequate sensitivity for relatively large effects, whereas small-to-moderate group differences may have remained undetected.

To examine the robustness of the primary finding, we conducted a univariate general linear model with V1–LOC connectivity as the dependent variable, group was entered as a fixed factor, while sex, age, education, and mean FD were included as covariates.

The group effect on V1–LOC connectivity remained significant after covariate adjustment, *F*(1, 59) = 30.809, *p* < 0.001, partial η^2^ = 0.343. Parameter estimates indicated that adjusted V1–LOC connectivity was significantly higher in the HC group than in the IGD group, B = 0.241, SE = 0.043, 95% CI [0.154, 0.328], *p* < 0.001. Sex, age, education, and mean framewise displacement were not significantly associated with V1–LOC connectivity (all *p* > 0.450).These results suggest that the reduced V1–LOC connectivity observed in the IGD group was not primarily attributable to demographic variables or head motion, as summarized in **Table 3**.

**Table 3:** Covariate-adjusted group effect on V1–LOC connectivity.

Effect	B	SE	95% CI	F	p	Partial η^2^
Sex, male vs. female	0.031	0.050	−0.068 to 0.130	0.399	0.530	0.007
Age	−0.009	0.012	−0.032 to 0.015	0.565	0.455	0.009
Education	0.009	0.017	−0.025 to 0.044	0.295	0.589	0.005
Mean FD	−0.216	0.485	−1.185 to 0.754	0.198	0.658	0.003
Group, HC vs. IGD	0.241	0.043	0.155 to 0.328	31.128	<0.001	0.342

V1–LOC connectivity was entered as the dependent variable. Group and sex were entered as fixed factors, and age, education, and mean framewise displacement were entered as covariates. Only main effects were included. IGD and female participants were treated as the reference categories in the parameter estimates. Therefore, the positive B value for group indicates higher adjusted V1–LOC connectivity in HCs than in individuals with IGD. Model R^2^ = 0.353, adjusted R^2^ = 0.298.

### 3.4 Associations between visual hierarchical measures and addiction severity

Within the IGD group, Pearson correlation analysis showed that V1–LOC connectivity was significantly negatively associated with IAT scores (*r* = −0.479, *p* = 0.004, FDR-corrected *p* = 0.016, *n* = 35). This association remained significant after controlling for age, sex, years of education, and mean framewise displacement (partial *r* = −0.447, *p* = 0.012, FDR-corrected *p* = 0.048, *df* = 29).

**Figure 3:**
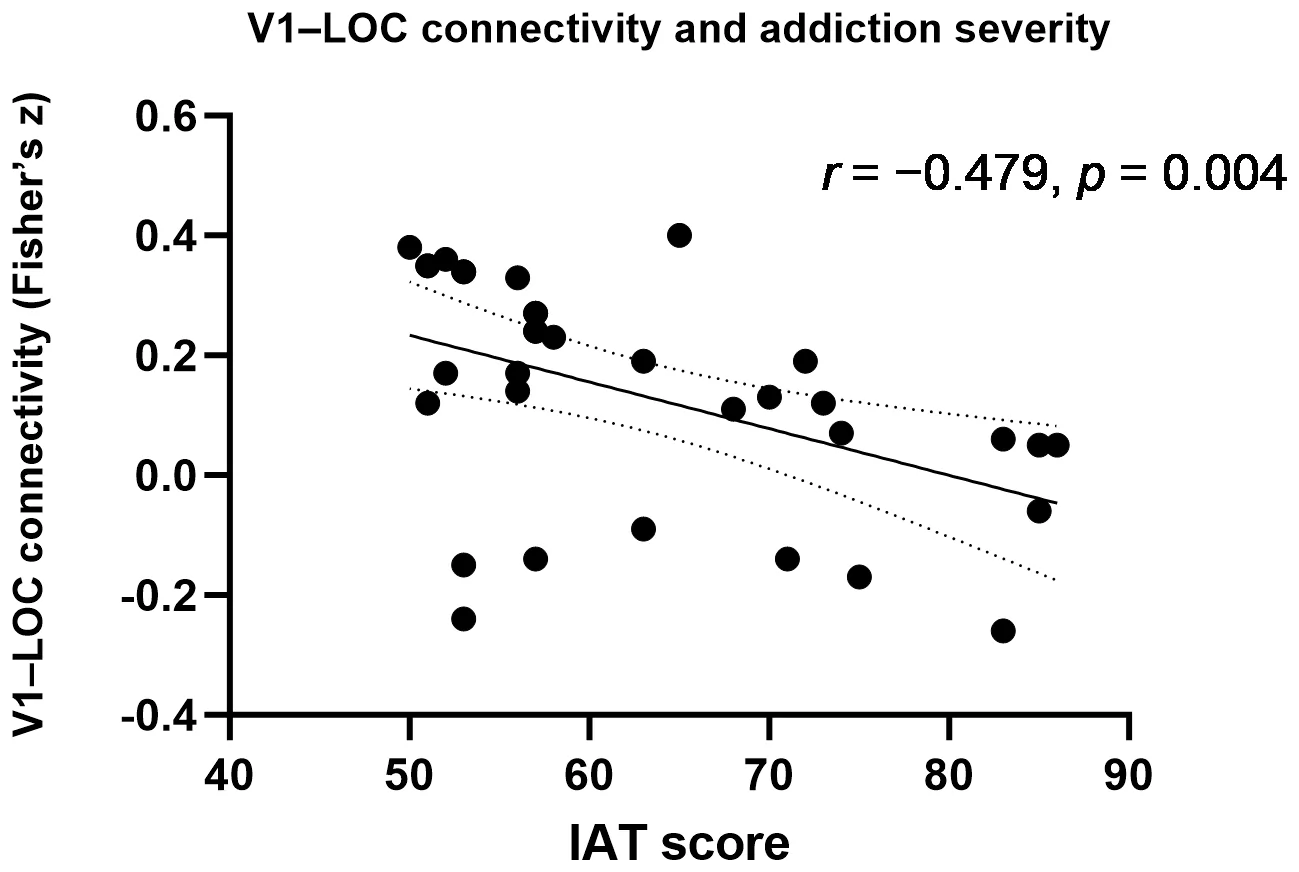
Association between V1–LOC connectivity and addiction severity in the IGD group. V1–LOC connectivity was the visual hierarchical measure showing a significant association with IAT scores after FDR correction.

To determine whether addiction severity was also associated with the other visual hierarchical measures, corresponding correlation analyses were performed for V1–Lingual connectivity, Lingual–LOC connectivity, and HII. None of these measures showed a significant association with IAT scores after FDR correction. Specifically, the partial correlations were −0.323 for V1–Lingual connectivity (*p* = 0.076, FDR-corrected *p* = 0.152), −0.058 for Lingual–LOC connectivity (*p* = 0.756, FDR-corrected *p* = 0.756), and 0.061 for HII (*p* = 0.745, FDR-corrected *p* = 0.756). The complete Pearson and partial correlation results are summarized in **Table 4**, and the V1–LOC association is illustrated in **Figure 3**.

**Table 4:** Associations between visual hierarchical measures and addiction severity in the IGD group.

Measure	Pearson r	*p*	FDR-corrected p	Partial r	*p*	FDR-corrected p
V1–Lingual	−0.301	0.079	0.158	−0.323	0.076	0.152
Lingual–LOC	0.008	0.966	0.966	−0.058	0.756	0.756
V1–LOC	−0.479	0.004	0.016	−0.447	0.012	0.048
HII	0.047	0.791	0.966	0.061	0.745	0.756

Correlation analyses were conducted within the IGD group (*n* = 35). Partial correlations were adjusted for age, sex, years of education, and mean framewise displacement (FD). *P* values were corrected across the four visual hierarchical measures using the Benjamini–Hochberg false discovery rate procedure.

### 3.5 Sensitivity analysis using alternative ROI sizes

To determine whether the main findings were influenced by ROI size, we repeated the primary ROI-based functional connectivity analyses using smaller spherical ROIs with radii of 5 mm and 4 mm, while maintaining the same MNI coordinates and analytical procedures as the primary 6-mm ROI analysis. The reduced V1–LOC connectivity in the IGD group remained significant when alternative ROI radii of 5 mm and 4 mm were applied (5 mm: *F* = 33.139, *p<* 0.001; 4 mm: *F* = 29.038, *p<* 0.001), with comparable effect sizes. Similar results were obtained across ROI sizes, indicating that the observed V1–LOC alteration was robust to different ROI definitions. Furthermore, the negative association between V1–LOC connectivity and IAT scores remained significant in partial correlation analyses across different ROI definitions (5 mm: partial *r* = −0.420, *p* = 0.019; 4 mm: partial *r* = −0.404, *p* = 0.024). Detailed results of the ROI-size sensitivity analyses are provided in **Supplementary Tables S4**, **S5**.

## 4 Discussion

The present study investigated intrinsic hierarchical functional organization within the visual system in IGD using a three-level model comprising the primary visual cortex (V1), lingual gyrus, and lateral occipital cortex (LOC). V1–LOC connectivity was significantly reduced in individuals with IGD compared with healthy controls, and this difference remained significant after FDR correction. By contrast, adjacent-level connectivity, including the V1–Lingual and Lingual–LOC pathways, did not show significant group differences. The hierarchical integration index (HII) was numerically lower in the IGD group, but this effect did not reach statistical significance. Within the IGD group, lower V1–LOC connectivity was associated with higher IAT scores. The MANCOVA further supported an overall alteration of visual hierarchical connectivity in IGD, while follow-up analyses indicated that this effect was primarily attributable to reduced V1–LOC connectivity. Overall, these findings suggest that IGD-related visual-system abnormalities may be most pronounced in long-range cross-hierarchical connectivity between early visual cortex and higher-order lateral occipital cortex. Rather than indicating a generalized reduction across all examined visual pathways, the present results highlight a statistically robust reduction in V1–LOC connectivity.

The reduction in V1–LOC connectivity offers a hierarchical perspective on visual processing abnormalities in IGD. Visual object perception requires the transformation of low-level visual features into higher-order representations that support stable and behaviorally meaningful perception. Recent theories of human object vision suggest that object representations do not arise from a single isolated region. Instead, they emerge from distributed and interacting visual systems that support multiple perceptual and behavioral goals (31). Converging evidence from computational and neurophysiological studies further indicates that recurrent interactions within the ventral visual stream are important for robust object recognition, especially when visual inputs are complex or behaviorally demanding (32–34). In this context, V1 mainly supports early visual feature encoding, whereas LOC contributes to higher-order object-related visual representation. The observed reduction in V1–LOC connectivity may therefore reflect less efficient communication between early sensory encoding and higher-order visual representation in IGD.

The lack of significant group differences in V1–Lingual and Lingual–LOC connectivity is also informative. This pattern suggests that IGD-related visual abnormalities may not reflect a diffuse disruption across the entire visual pathway. Rather, adjacent-level coupling within the examined hierarchy may be relatively less affected, whereas long-range bridging between early visual cortex and higher-order visual association cortex appears more vulnerable. The HII was numerically lower in the IGD group and showed the same direction of change as the V1–LOC finding, but it did not reach statistical significance. HII should therefore be interpreted cautiously because it did not provide statistically significant evidence for altered hierarchical visual integration. This may be partly because HII integrates information from both long-range V1–LOC connectivity and local V1–Lingual coupling. When local coupling is relatively less affected but long-range connectivity is reduced, the composite index may show a smaller overall effect and lower statistical sensitivity.

Reduced long-range cross-hierarchical connectivity may be associated with differences in processing of gaming-related visual cues. Online games provide highly dynamic, visually complex, and reinforcement-rich stimuli. Prolonged exposure to such environments could bias visual processing toward rapid local sensory encoding and increase the need for coordination between early visual areas and higher-order visual association cortices. When V1–LOC communication is reduced, local visual features may still be processed, but their integration into stable higher-order visual representations may become less efficient. This imbalance may be associated with altered processing of gaming-related visual information. during perception and attention. In turn, these features may more readily contribute to attentional capture and abnormal salience attribution, consistent with broader theories of attentional bias and incentive sensitization in addictive behaviors (35, 36).

Previous studies provide converging support for the role of gaming-related visual cues in IGD. Electrophysiological evidence has shown enhanced event-related responses to game-related visual cues in individuals with IGD, suggesting increased attentional bias toward gaming stimuli (37). Cue-reactivity research further indicates that even distal gaming-related cues can induce craving responses in individuals at risk for gaming disorder (38). Functional neuroimaging studies have also reported that gaming cue-reactivity in IGD is accompanied by alterations in large-scale functional networks (39). Together, these findings suggest that abnormal sensory and attentional processing of gaming-related visual cues may contribute to the neural mechanisms of IGD.

This interpretation is also consistent with the I-PACE framework. The I-PACE model proposes that specific Internet-use disorders arise from interactions among individual predispositions, affective and cognitive responses to disorder-related cues, cue-reactivity, craving, and diminished executive control. From this perspective, gaming-related visual cues must be processed through sensory systems before they can elicit attentional bias, cue-reactivity, or gaming urges. Reduced V1–LOC connectivity may therefore reflect a sensory-level neural abnormality in IGD. Specifically, gaming-related visual information may be processed in a more local and highly salient manner, while its integration into higher-order visual representation systems may become less efficient. This visual hierarchy perspective complements existing neuroimaging literature on IGD, which has primarily emphasized large-scale networks and brain systems related to reward, salience, and executive-control functions (40, 41).

Broader addiction research also highlights the role of visual-network dysfunction in addiction-related cue processing. Visual cortical activation is a consistent component of cue-reactivity in addiction, and sensory systems may contribute to addictive behaviors beyond classical reward and control circuits (11, 12). Addiction-related cues can also capture attention through stimulus-driven mechanisms, especially when salience attribution and inhibitory control systems are disrupted (20, 21). Recent findings from tobacco use disorder further suggest that visual networks may interact abnormally with salience and executive-control networks. This pattern has been described as a hierarchical dysregulation involving altered top-down control and disrupted bottom-up processing (42). Although this evidence comes from substance-related addiction rather than IGD, it supports the broader involvement of visual-network dysregulation in addictive disorders and provides a useful comparative framework for interpreting the present V1–LOC finding.

The negative correlation between V1–LOC connectivity and IAT scores further suggests the clinical relevance of this long-range visual pathway. Within the IGD group, lower V1–LOC connectivity was associated with greater addiction severity. This association links the group-level connectivity alteration to individual differences in symptom burden.

More broadly, addiction has been associated with abnormal salience attribution, impaired inhibitory control, and a shift from voluntary behavior toward more habitual or compulsive patterns of responding (21, 43). In IGD, reduced integration between early visual input and higher-order visual representation may interact with these broader addictive processes. Such interaction could reflect a neural correlate associated with individual differences in symptom severity. Nevertheless, this correlation should be interpreted cautiously. It suggests potential clinical relevance but does not establish V1–LOC connectivity as a diagnostic biomarker, which would require larger samples, independent validation, and predictive modeling.

## 5 Limitations

Several limitations should be noted. First, the present study used a simplified three-level visual hierarchy model comprising V1, lingual gyrus, and LOC. Although this hypothesis-driven ROI approach allowed us to examine long-range visual hierarchical connectivity in a focused manner, it may not fully capture the fine-grained organization of the ventral visual pathway or the broader visual network. Future studies using more detailed visual parcellations, retinotopic mapping, or whole-network approaches are needed.

Second, the sample was recruited from a single center and was relatively modest in size. The sensitivity power analysis indicated that the current sample could reliably detect relatively large between-group effects (approximately Cohen’s *d* ≥ 0.71), whereas smaller effects may have remained undetected. Larger independent cohorts are therefore required to validate the findings and improve sensitivity to small-to-moderate effects.

Third, this study was based on resting-state fMRI and did not directly examine responses to gaming-related visual cues. Resting-state functional connectivity reflects statistical coupling between regional time series rather than directed causal influence (44). Future studies combining resting-state and task-based cue-reactivity paradigms may clarify how V1–LOC connectivity is involved in cue-evoked visual processing.

Finally, the cross-sectional design limits conclusions about causality or developmental trajectory. Longitudinal studies are needed to determine whether reduced V1–LOC connectivity reflects a pre-existing vulnerability, a consequence of prolonged gaming exposure, or a change associated with symptom progression or remission.

Although mediation analysis could theoretically examine whether V1–LOC connectivity statistically links diagnostic status and symptom severity, the current sample size and cross-sectional design limit the reliability of such causal modeling. Future studies with larger samples should investigate this possibility.

## 6 Conclusion

In conclusion, the present study suggests that IGD is associated with altered intrinsic hierarchical organization within the visual system. Using an *a priori* three-level visual hierarchy model comprising V1, lingual gyrus, and LOC, we found significantly reduced long-range V1–LOC functional connectivity in individuals with IGD, whereas adjacent-level V1–Lingual and Lingual–LOC connectivity did not show significant group differences. Within the IGD group, lower V1–LOC connectivity was associated with greater addiction severity. These findings suggest that IGD-related visual abnormalities may be most prominent in long-range cross-hierarchical communication between early visual cortex and higher-order lateral occipital cortex. This visual hierarchy perspective may complement existing reward- and control-centered accounts of IGD and provide a sensory-level framework for understanding maladaptive processing of gaming-related visual cues.

## Data Availability

The raw data supporting the conclusions of this article will be made available by the authors, without undue reservation.
