# Supplementary material for: Altered long-range visual hierarchical connectivity in internet gaming disorder: reduced V1–LOC connectivity and its association with addiction severity
Source: Front Psychiatry. 2026 Sep 11;17:1883842. doi: 10.3389/fpsyt.2026.1883842 (PMC13613021; doi:10.3389/fpsyt.2026.1883842)
Supplement: Supplementary file 1 [file DataSheet1.pdf]

Supplementary Table S1. Anatomical rationale for visual hierarchy ROIs

ROI	MNI coordinates	Visual level	Anatomical rationale
V1	L [−10, −80, 8]; R [10, −80, 8]	Early visual level	Located in the bilateral calcarine cortex/primary visual cortex region
Lingual gyrus	L [−14, −60, −2]; R [14, −60, −2]	Intermediate visual level	Located in the bilateral lingual gyrus, an intermediate medial occipitotemporal visual region
LOC	L [−44, −72, −4]; R [44, −72, −4]	Higher-order visual level	Located in the bilateral lateral occipital cortex, a higher-order object-related visual region

Note. ROI coordinates were defined a priori in MNI space to sample representative locations within early, intermediate, and higher-order visual processing regions.

Anatomical localization was visually inspected on the MNI template to confirm that the spherical ROIs were located within the intended visual regions.

Supplementary Table S2. Follow-up univariate analyses of ROI-based visual hierarchical functional connectivity after MANCOVA.

Connectivity measure	F value	p value	FDR-corrected p value	Partial η^2
V1–Lingual	4.046	0.049	0.073	0.064
Lingual–LOC	2.857	0.096	0.096	0.046
V1–LOC	30.809	<0.001	<0.001	0.343

Note. F values were derived from follow-up univariate analyses after MANCOVA. Age, sex, years of education, and mean framewise displacement (FD) were included as covariates. P values were corrected across the three follow-up univariate tests using the Benjamini–Hochberg false discovery rate procedure.

Supplementary Table S3. Multivariate analysis of covariance for visual hierarchical connectivity

Effect	Wilks' λ	F	df1	df2	p	partial η²
Group	0.656	9.95	3	57	<0.001	0.344
Sex	0.943	1.142	3	57	0.340	0.057
Age	0.895	2.233	3	57	0.094	0.105
Education	0.936	1.301	3	57	0.283	0.064
mean FD	0.967	0.656	3	57	0.582	0.033

Note. MANCOVA included V1–Lingual, Lingual–LOC, and V1–LOC connectivity as dependent variables. Group was entered as the fixed factor, while age, sex, years of education, and mean framewise displacement (FD) were included as covariates. HII was not included because it was mathematically derived from V1–LOC and V1–Lingual connectivity. Wilks' λ, Wilks' lambda; FD, framewise displacement; partial η², partial eta squared.

Supplementary Table S4. Group differences in V1–LOC connectivity using alternative ROI sizes.

ROI size	Connection	HC (mean ± SD)	IGD (mean ± SD)	F value	p value	Partial η²
5 mm	V1–LOC	0.3377 ± 0.1364	0.1140 ± 0.1760	33.139	<0.001	0.360
4 mm	V1–LOC	0.2620 ± 0.1480	0.0860 ± 0.1612	29.038	<0.001	0.330

Note. Group differences were assessed using the same covariate-adjusted statistical procedure as in the primary 6-mm ROI analysis, with age, sex, years of education, and mean framewise displacement (FD) included as covariates. Partial η² represents the effect size of the group effect. HC, healthy controls; IGD, Internet gaming disorder; LOC, lateral occipital cortex; ROI, region of interest; FD, framewise displacement.

Supplementary Table S5. Partial correlations between V1–LOC connectivity and IAT scores across ROI sizes.

ROI size	Participants	r	p value
6 mm (primary analysis)	IGD	-0.447	0.012
5 mm	IGD	-0.420	0.019
4 mm	IGD	-0.404	0.024

Note. Partial correlations were adjusted for age, sex, years of education, and mean framewise displacement (FD). IGD, Internet gaming disorder; IAT, Internet Addiction Test; LOC, lateral occipital cortex; ROI, region of interest.
